# IMSC-EIoTD: Identity Management and Secure Communication for Edge IoT Devices

**DOI:** 10.3390/s20226546

**Published:** 2020-11-16

**Authors:** Kazi Masum Sadique, Rahim Rahmani, Paul Johannesson

**Affiliations:** Department of Computer and Systems Sciences, Stockholm University, 16407 Stockholm, Sweden; pajo@dsv.su.se

**Keywords:** IoT, identity management, IoT security, authentication, authorization, edge computing, fog computing, decentralized, trust, context-aware computing, trusted computing platform

## Abstract

The Internet of things (IoT) will accommodate several billions of devices to the Internet to enhance human society as well as to improve the quality of living. A huge number of sensors, actuators, gateways, servers, and related end-user applications will be connected to the Internet. All these entities require identities to communicate with each other. The communicating devices may have mobility and currently, the only main identity solution is IP based identity management which is not suitable for the authentication and authorization of the heterogeneous IoT devices. Sometimes devices and applications need to communicate in real-time to make decisions within very short times. Most of the recently proposed solutions for identity management are cloud-based. Those cloud-based identity management solutions are not feasible for heterogeneous IoT devices. In this paper, we have proposed an edge-fog based decentralized identity management and authentication solution for IoT devices (IoTD) and edge IoT gateways (EIoTG). We have also presented a secure communication protocol for communication between edge IoT devices and edge IoT gateways. The proposed security protocols are verified using Scyther formal verification tool, which is a popular tool for automated verification of security protocols. The proposed model is specified using the PROMELA language. SPIN model checker is used to confirm the specification of the proposed model. The results show different message flows without any error.

## 1. Introduction

**Figure 1:**
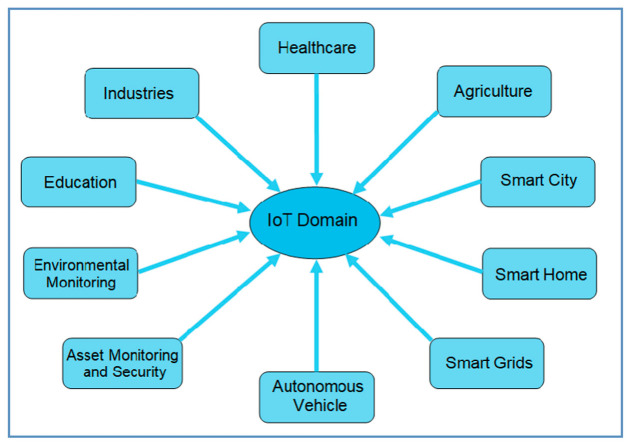
Representation of the IoT domains.

The Internet of things (IoT) is a large-scale paradigm. In the IoT paradigm devices communicate with each other regardless of their owners. The communication is not only between machines to humans but also between machine to machine [1] and machine to smart objects [2]. The Internet of connected devices namely the Internet of things (IoT) includes different domains (shown in Figure 1) like healthcare, agriculture, smart city, smart home, smart grid, automated vehicles, assets monitoring, environmental monitoring, education, industries, and so on [3]. Each of the above-mentioned domains includes a huge number of sensors, actuators, gateways, servers, and related end-user applications [1,3,4]. The data collected from the interconnected objects or things are used for producing results in different IoT applications. IoT applications are enhancing our society as well as our daily life with different innovative services [4]. The fundamental blocks of any IoT solution are connected devices, communication networks, services, management, security, and applications [1,5]. IoT devices need to be self-configurable, adaptable with the environment, and will have a unique identity for accessing over the internet [5]. The distribution of heterogeneous IoT devices is increasing every day. Traditional internet security algorithms are not appropriate for the IoT paradigm due to the heterogeneous and dynamic behavior of large numbers of IoT devices [2,6]. New dynamic protocols and algorithms are required to address new security threats as a large number of IoT devices are deployed with accessibility from anywhere in the world. IoT devices need to authenticate before connecting or transferring data between each other [6]. The authentication of IoT devices is required for all the IoT application domains: healthcare, smart grids, smart cities, autonomous vehicles, industrial IoT, etc. [7].

To ensure the authentication and authorization, identification of each IoT device is crucial. Fog/edge computing is becoming popular in the deployment of IoT applications because of its distributed architecture and availability near to the source of information [8]. IoT security management at the Fog layer is also becoming popular as it is easy and more effective to detect and isolate victim IoT devices that are being compromised due to malicious attacks [8,9]. IoT devices are developed by different vendors without standardization which hinders the assurance of trust and security between different IoT devices [1,10]. The existing IPv4 or IPv6 based identity management and authentication solutions are not suitable for the future IoT paradigm [11]. There is no standardized or decentralized identity management system for the future adoption of huge IoT devices to be authenticated for communication with relevant applications [8].

The contributions in this paper are listed below:

- A detailed analysis of the current state of identity management for the IoT
- A decentralized edge-fog based identity management architecture is proposed for edge IoT devices and gateways.
- A new authentication mechanism for IoT devices is proposed with dynamic authorization and/or access control mechanism near the edge IoT devices where access to new resources is granted, and activities are recorded with the context information.
- A new secure communication protocol is proposed for edge IoT devices and IoT gateways and it is verified using the Scyther security verification tool.
- The proposed architecture is verified and simulated using the SPIN verification tool.

The rest of the paper is organized as follows: in Section 2 we have discussed the background and motivation of this research, where we have briefly elaborated related terms and presented the research problem to create a common understanding among the authors and readers. In Section 3, the related works are discussed. In Section 4, we have presented our proposed model followed by results and discussion in Section 5. Section 6 concludes the paper with a discussion for future research directions. In this paper, we have tried to address readers from beginners to experienced scholars. As a result, the paper could seem to be lengthy for readers. So, we suggest the following road map for readers. A beginner reader may read each section in sequential order while an expert reader can directly go to Section 4, which is our proposed solution.

## 2. Background and Motivation

As discussed in the above section, innovative services based on the internet of things (IoT) not only improve human living but also improve industrial production, supply chain management, employees working efficiency at offices, educational processes, and so on [4]. As the IoT paradigm is built upon smart connected heterogeneous objects, the objects need to be identified in a way that improves data analysis experiences as well as reduces the risk of miss leading results based on data collected from a compromised IoT object [1]. IoT devices need to be authenticated based on identity information. After the authentication process, the authorization takes place. The identity management of heterogeneous devices is still an open challenge [1,8,11,12]. Motivated by this we have proposed a novel model for identity management of the internet of things (IoT) devices. In our proposed model, we have also described how to secure data communication between IoT devices and the corresponding gateways. Before describing our proposed model, we will discuss some relevant terms which will lead the readers to easily understand our model.

### 2.1. IoT Identity Management

Identity management is the process of door keeping in which we save information of individual users or devices in a database for further verification when it is needed. Identity management of IoT devices is different from the traditional identity management of users because of its heterogeneous behavior with mobility properties [12]. As the IoT paradigm is enhanced every day with new use cases, the need for identity management also increases [13]. The innovative IoT applications need an IoT infrastructure where IoT devices are owned by someone, managed by someone, maintained by someone, and used by huge communities. We are talking about an IoT platform where different IoT devices, different IoT applications, and different IoT users interact with each other. The identity information of devices is important as it may happen that different user applications communicate and data transfer with different end devices [12].

### 2.2. IoT Authentication

Authentication is the process of verification of the identity of any person or device who claims himself as someone [8]. IoT device authentication could be different from the traditional authentication of users. A traditional authentication of a user of an application in a digital world is mostly based on a username and a password or using pre-shared secrets [13]. Token and secret based authentication is another type of user authentication process which is more related to the enhancement of authorization process. An IoT authentication process needs to be smart enough to detect a compromised device dynamically based on context and/or the behavior of that device even though the device was previously authenticated. It means in an authentication process for IoT devices any authenticated device may be detected as unauthenticated based on the data it produces or transfers [14,15]. So, an IoT authentication process is much more complex as it is related to the context in which the device is authenticated. An authenticated IoT device can be unauthenticated based on the contextual data it produces.

### 2.3. IoT Authorization

Authentication and authorization are tightly coupled with each other in the digital world. The authorization always comes after the authentication. In authentication, identity verification is performed and in authorization, verified entities get access to a single or a group of resources. Authorization is the process of granting access to a resource based on the rights of a user or a machine. The information on the rights is documented in a database that is used for validation of the rights based on a request to access a resource by any specific entity. Only authenticated and authorized entities get access to a specific resource it requests for access [16]. Based on the use case and context, the IoT authorization process could be also different. IoT devices and applications may communicate and collaborate frequently and the authorization process needs to be dynamic. In the traditional authorization process, the authorization is performed based on authorization policies saved earlier in a database. However, in IoT authorization, the authorization process could be dynamic based on the instance request from the IoT device or IoT application [17]. In a dynamic authentication process, context information plays a vital role. In case a malicious application or malicious user tries to access a certain resource, the context information [18] can be used to validate this request.

### 2.4. IoT Communication Process

The Internet of things (IoT) is built upon communication networks. The IoT communication processes include communication between different IoT devices, IoT devices and IoT gateways, IoT devices to and from end-user applications, IoT devices to servers, and so on. IoT communication process needs to be secure [19]. The IoT components communicate with each other based on needs, for example, real-time data processing, where traditional authentication via a centralized [15] server is not efficient. IoT communications take place within the IoT architecture, which is described in detail in Section 2.6. The IoT data needs to be collected from the sensor networks and further processed before it is stored in the servers. The following section described the IoT data collection, aggregation, and processing models.

### 2.5. IoT Data Aggregation and Processing Models

IoT data aggregation is the process of collecting raw data from different sensors before it is transferred to the sinks for further analysis [6,20]. In a distributed data aggregation process, distributed algorithms are used to avoid data conflicts and time conflicts [20]. IoT device identification and authentication are also essential for data aggregation because data from the authenticated sources should only be considered [21]. After the aggregation of raw data from different sensor devices, data is further processed for different IoT applications. Data reliability, scalability, interoperability are emerging challenges in the IoT data processing due to diversity and heterogeneity of IoT devices. IoT Data processing is required for knowledge acquisitions [22]. IoT data can be processed in centralized [15], decentralized, and distributed [15,23] manners. The identity management of IoT devices, IoT authentication, and authorization can also be implemented using centralized or decentralized models. Below we have discussed centralized, decentralized, and distributed data processing highlighting IoT authentication, authorization, and identity management.

#### 2.5.1. Centralized Data Processing Model

In a centralized data processing model, IoT data collected, and aggregated from the heterogeneous sensor networks is sent to the centralized server for further analysis [15]. Centralized data processing is becoming less popular, due to the requirement of real-time data processing of emerging IoT use cases, which is not possible in a centralized data processing model. For example, in smart healthcare service, quick decision making is essential [15]. Additionally, for industrial IoT use cases and autonomous vehicles, short time decision making is worthy. As the IoT data processing use cases are moving toward the source of data, it is necessary to have source identity and authentication mechanisms near to the IoT data sources.

#### 2.5.2. Decentralized and Distributed Data Processing Model

In a decentralized and distributed [15,23,24,25] model IoT data is processed near to the source of data using distributed algorithms. The decentralized [26] data processing is required when group-level decision making is also essential besides the real-time data processing at the local edge/fog devices. For example, at a busy city local decision making is needed for local traffic management and on the other hand, group-communication and collaboration are required for route planning to improve the situation of traffic jams and to suggest the best routes to the end-users of IoT applications. Secure communication, identity management with authentication, and authorization are still valid for all these innovative IoT use cases. We have described IoT architecture and different IoT related computing paradigms below.

### 2.6. IoT Architecture

To enhance the IoT security, different researchers proposed different layered architecture for IoT: some researchers proposed three-layered architecture, a few researchers proposed four-layered architecture, and a few researchers proposed five-layered architecture. The most common architecture has three layers: application layer, network layer, and perception layer [27]. In this architecture, the application layer is where the data is stored, analyzed and represented as knowledge, the network layer is where the data is transferred via different network devices, and the perception layer is where the data is generated, collected, aggregated and processed before it is transferred to the network layer. The main drawback of this architecture is that it is very abstract for the implementation of security and enhancement of service quality for the emerging IoT applications.

**Figure 2:**
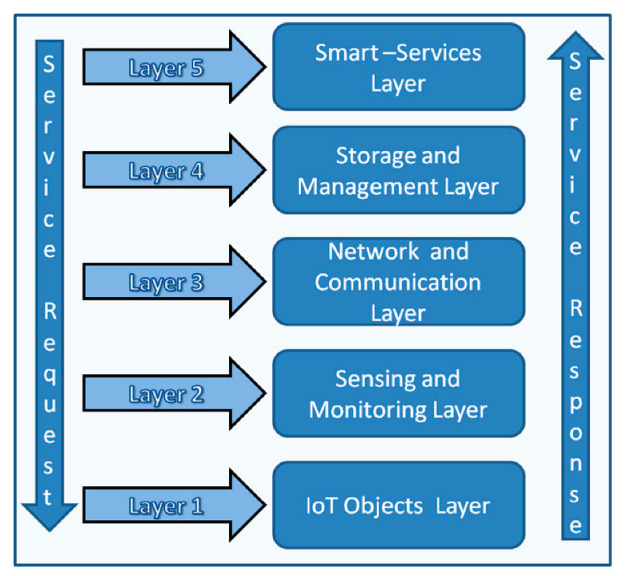
Service-oriented five layered representation of IoT architecture.

Influenced by the above-mentioned drawbacks of IoT architectures, a five-layered service-oriented IoT architecture (Figure 2): layer 1 (IoT Objects Layer), layer 2 (Sensing & Monitoring Layer), layer 3 (Network & Communications Layer), layer 4 (Storage and Management Layer), and layer 5 (Smart-Services Layer) was proposed and discussed in our previous papers [28,29]. Identity management and authentication of IoT devices near to the source (Fog/Edge Layer) is essential to ensure security at layer-1, layer-2, and layer-3 of the proposed IoT architecture. The identity of IoT end devices is also important for storing and managing data at layer-4 and in smart services at layer-5 to ensure the source of data. Currently, IoT data is mostly transferred to cloud devices. In the subsequent sections, we have discussed the IoT application layer, cloud computing layer, fog computing layer, edge computing layer, and IoT end device layer which is worthy to understand our proposed model.

#### 2.6.1. IoT Application Layer

IoT application layer is the top layer of the IoT paradigm. It is the smart-service layer in the service-oriented architecture presented in Figure 2. Data collected from the end IoT devices are processed at different IoT layers and are presented as information in the end-user applications [28]. In a centralized cloud-centric model, IoT data is always presented to the IoT applications via the cloud computing layer. As the need for real-time and quick decision making for the IoT applications is increasing the need for distributed decentralized IoT architecture is increasing. In a decentralized distributed IoT architecture the IoT applications get results from the edge computing or fog computing layer which reduces the network traffic as well as increases the efficiency of the application.

#### 2.6.2. Cloud Computing Layer

**Figure 3:**
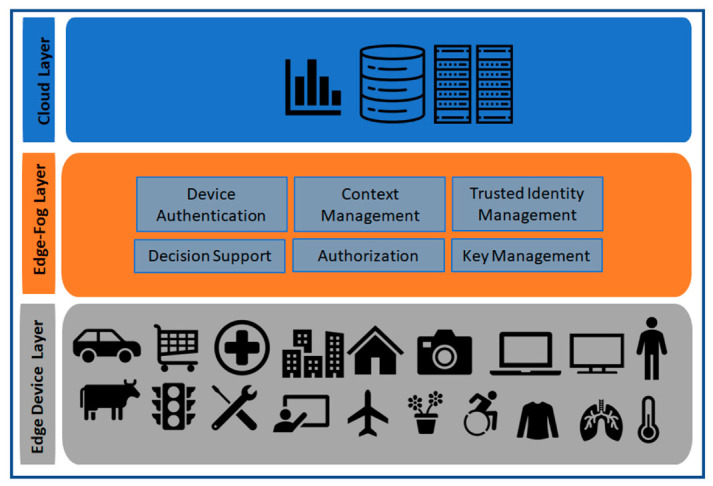
IoT trust management at edge-fog layer (adapted from reference [9]).

Cloud computing is the most popular IoT data processing platform. It is presented as the storage and management layer in the service-oriented architecture presented in the Figure 2 and presented as cloud layer in the Figure 3. The IoT devices communicate with the cloud computing devices in a centralized manner and transfer data to the cloud devices for centrally processing, storing, and further analysis before the data becomes available to the IoT end applications. Cloud computing is also popular for unlimited resources and high processing capability with flexibility and scalability [30]. Recently proposed IoT identity management solutions are mostly cloud-based centralized models [31,32]. Though the centralized cloud based IoT architecture has many advantages, it is not feasible for IoT use cases where real-time data processing and quick decision making with low latency and less delay are worthy [30]. The layer down to the cloud computing layer is fog computing layer (see Figure 3), which is described below.

#### 2.6.3. Fog Computing Layer

The fog computing concept was proposed by Cisco [30,33,34,35,36,37,38]. Fog computing is also called edge computing by many researchers. Fog Computing layer is the middle layer for internet communication for any connected device which is involved in data collection and analysis. It is the sensing and monitoring layer in the service-oriented architecture presented in the Figure 2, and as Edge-Fog layer in the Figure 3. The fog computing brings partly the cloud facilities near to the edge of the network. Fog computing devices have more storage capacity and computational abilities than edge devices but less than the cloud devices. The fog layer is connected to the cloud layer via a high-speed internet connection. Fog layer can quickly respond to the edge devices and can process the IoT data quickly which is the main advantage of the fog computing layer. A quick analysis of security variability can be possible at the fog layer, fog layer identity management, and authentication can improve the performance of the IoT paradigm as the Fog layer is closer to the source of IoT data and has more computational power than the IoT edge devices. The layer down to the fog layer is the edge layer, which is described below.

#### 2.6.4. Edge Computing Layer

The edge computing [26,30,33,34,38] is the nearest layer which can provide computing capabilities to the end devices. The upper layer of the edge layer is the fog computing layer and the lower layer is the end device layer. As the edge layer and fog layer are close to each other, it is also presented as sensing and monitoring layer in the service-oriented architecture in the Figure 2, and as Edge-Fog layer in the Figure 3. Edge gateways and edge network communication devices are the main component of the edge computing layer. Sometimes the edge computing and the edge IoT device layer are presented at the same layer because edge gateway devices are directly connected with the IoT end devices but have better computational capabilities than the IoT end devices (sensors and actuators). Edge computing can play a vital role in the enhancement of IoT device authentication and IoT security at IoT end devices because end IoT devices are small resource-constrained devices (computation, memory, storage) and mostly battery-driven. However, the edge gateways, routers, switches are connected to a constant power resource and have more computational capabilities.

#### 2.6.5. IoT End Device Layer

IoT end devices are at the lower layer of the IoT paradigm. IoT sensors and actuators are the main components of this layer. Some researchers also call this layer as the perception layer [27]. It is presented as the IoT objects layer in the service-oriented architecture presented in the Figure 2 and presented as edge device layer in the Figure 3. Raw data are collected from this layer to the nearest IoT gateway for further processing. Sometimes end IoT devices are also called edge devices as it is at the edge of the network. The security of IoT data is crucial but challenging as this layer is built upon resource-constrained devices. Implementations of heavyweight security algorithms are not feasible at these resource-constrained devices due to computation, memory as well as power resource limitations. In our proposed model we have considered identity management of these end devices and proposed secure data transfer between authenticated devices using lightweight encryption algorithms.

### 2.7. IoT Trust Management Model

Trust is a concept that leads to a level of interactions between two or more entities. Trust management in IoT is essential for communication, and data exchange between different entities of the IoT paradigm [39]. IoT devices communicate with each other based on the applied network topology.

The Figure 3 represents a trust management model at edge-fog layer of IoT paradigm. The IoT devices need to be authenticated [40] and only trusted entities should be able to collaborate. Sensitive data should be transferred only to a trusted entity that should be handled by an access control/authorization mechanism. Though there are cloud-based identity solutions by different business providers, a cloud-based centralized identity management solution is not feasible for many of the use cases described above. To handle authentication and authorization of IoT devices we need an identity management solution near to the devices. Motivated by these and to solve the above-mentioned problems, in this paper, we have proposed a new decentralized identity management model for edge IoT devices. Before describing our proposed solution, we have discussed the related work below.

## 3. Related Works

Recently, many researchers are addressing identity management for components of the IoT network. Cloud-based identity verification and management are discussed in [31,32]. In [31] the authors have described Security Assertion Markup Language (SAML) based representation of existing IoT identity management solutions. The authors also compared the SAML based JSON and XML representations of identity information. They have also developed prototypes for testing their proof of concept. Cloud-based federated identity management via API is proposed in [32]. The proposed model is valid for IoT devices and users. The authors have discussed the interoperability of different IoT service providers. As we have discussed earlier, cloud-based centralized identity management solutions are not feasible for many IoT use cases and localization of identity management systems is important.

Secure access control and identity management mechanisms are proposed for Smart Grids in [41]. The authors have proposed a time-based one-time password (TOTP) based authentication model with three major system components. The components of their model are the central system, the data concentrator, and the smart meter. The communication between the central system and the data concentrator is based on public-key encryption and the communication between the data concentrator and the smart meter is symmetric-key encryption [41]. The authors also proposed a role-based access control for the users to ensure proper authorization to the resources. The main drawback of this model is that all the cardinals are handled by a central system, which opens the risk of a single point of failure in case the central system is compromised or unable to handle requests for certain failure. Additionally, this type of method can be only applicable to the specific use case of a smart grid solution and not much appropriate as a generic solution for identity management for IoT devices.

Different researchers have addressed the user-centric authentication in the IoT infrastructure [42,43]. In [42], authors have proposed a seven-phase smart card authentication model for user authentication and key exchange between the servers with multiple instances. The solution is focused on communication between wearable devices and mobile devices of users, but our research focus is a generic identity management solution for heterogeneous IoT devices. Identity solutions using smart cards could be more feasible for user-centric solutions where user identity is a major concern but not a feasible solution for heterogeneous IoT devices. User-centric identity management is proposed in [43]. The contributions in this work are a universal resource locator (URL) type identity suggestion for user devices. In this model, the authors have described three major subsystems, the device subsystem, the service subsystem, and the identity provider subsystem. The main drawback of this model is that the authors have considered that IoT devices are connected and owned by end-users but in the current age, an IoT device owned by a third party can be accessed by users or other organizations.

An identity management model based on network resource ID fragmentation is proposed in [44]. The authors discussed a fog-to-cloud solution for the identity management of network resources. This concept has some basic similarities with our proposed model. We have also proposed fog layer identity management, which is near to the edge devices. However, the main difference between the solution in [44] and our model is we have proposed distributed decentralized identity management via IDMS servers at fog layer but in [44] the detail of the database management system of the IDMS solution is discussed. We have also discussed in detail how secure communication is performed between the edge nodes, edge gateways, and identity management server which is missing in [44].

The identity management of the Internet of Things (IoT) devices is in the attention of the researcher for more than a decade [45], yet there is no well-established solution for it. In [45], the authors have discussed a model namely Identinet and the digital shadow. In the Identinet model, every entity is presented by an identity. The concept of digital shadow is also presented as a virtual identity for logical nodes in the proposed infrastructure. The authors have discussed concepts of identity for everything, for example, identities for software, identities for services, identities for vehicles, identities for sensor clouds, identities for users, etc. Our proposed model also has basic similarities with the concept of identity for everything, but we have extended this concept further towards a fog-edge centric solution.

In [46], authors have proposed the Identity Federation solution for cellular-based Internet of Things (IoT) devices. The main assumption in this article is that all IoT devices should have Subscriber Identity Modules (SIM). According to the author’s description, the device identity should be verified via an identity management server. The identity management server connects with the one-time password generator server (OTP) for a one-time password generation for the IoT devices. Though our proposed concept has some similarities with the concepts presented in [46], our model is not influenced by this work, and our model is more generic and valid for cellular based as well as other internet solutions. We have also addressed the issue of localization of identity management near to the gateways and described how secure communication is possible in IoT infrastructure.

An identity management solution based on device pattern extraction is proposed in [47]. The authors have described extraction of different device behavioral information (communication distance, data transfer rate, communication delay) for a specific time using a device extractor and save it to a database for further identification of the device. Although this concept is unique, it is still not feasible for devices with mobility capability. Future IoT devices will have more mobility capabilities which will hinder identity management of IoT devices using the proposed method.

An identifier management with interoperability architectures is proposed in [48]. The authors have proposed a five-layered interoperability framework. The authors have also discussed the concept of local and global identity platform and described the operation of the model. Besides academic researchers, the identity management of IoT devices is also in the attention of different industrial researchers. This work discussed more on the localization of identifiers but didn’t describe in detail about the IoT device identity management itself nor verified the proposed model.

Ericsson’s researchers have discussed the need for identity management for IoT devices in their technical papers. They have also presented architectures for interoperability enhancement using a cross-domain identity management model [49]. Cross-domain identity management is required when devices hold different identity information within different domains. The authors described two different architectures in which different identity management servers communicate with each other via a coordinating IDMS server or the directly IDMSs communicate with each other. The main drawback of this work is an extensive security analysis of the proposed IDMS architecture is not present.

Though the authors in [48,49] have presented a distributed architecture for identity management systems for the internet of things they still didn’t extend it with practical details on how the secure communication between the IDMS servers and the IoT devices will be performed. Additionally, in [48,49], authors did not verify their proposed model. However, in our proposed model we have discussed the concept of inter IDMS communication, management of security cardinals, followed by simulation results. We have proposed an edge-fog based model with localization of IDMS servers near to the IoT devices and the coordination between IDMS in our model is done via the cloud IDMS server. We have also suggested secure communication not only between the IoT devices and the gateways but also secure communication between the IoT device and the identity management server during the identity assignment process.

Identity management of large numbers of heterogeneous IoT devices is still an open issue. Different researchers tried to address different issues related to identity management for components of the internet of things (IoT) paradigm. The previous studies showed that the identity management of heterogeneous IoT devices with different types of network and communication processes is a big challenge. Cross-domain interoperable identity management is also another big challenge. In our research, we have tried to address the challenges found in previous studies and proposed an edge-fog based generic identity management model for edge IoT devices. We have also presented a new secure communication protocol for IoT end devices and IoT gateways. Our proposed model is presented in next section.

## 4. Proposed Model

This section presents our proposed identity management and secure communication model. The proposed identity management system consists of distributed identity providers which store and share identities and security cardinals with the edge IoT devices and edge IoT gateways. The model has four parts: IoT Devices (IoTD) at the edge IoT device layer, Edge IoT Gateway (EIoTG) at the edge computing layer, Local Identity Provider (LIdP) at the fog/edge layer, and Global Identity Provider (GIdP) at the cloud layer. The rest of this section is organized as follows: Section 4.1 presents the ontology of identity management, authentication, and authorization related to our proposed model, Section 4.2 presents notation of different terms used in the proposed model, assumptions, detailed working principles of the new IoT identity management system with descriptions of secure IoT communication protocols for the IoT paradigm, Section 4.3 describes the primitive finite state machine (pFSM) representations of logical interactions between different parts of our proposed model. Finally, in Section 4.4 we have described our proposed secure communication protocol for the IoT paradigm.

### 4.1. IoT Identity Management Ontology

**Figure 4:**
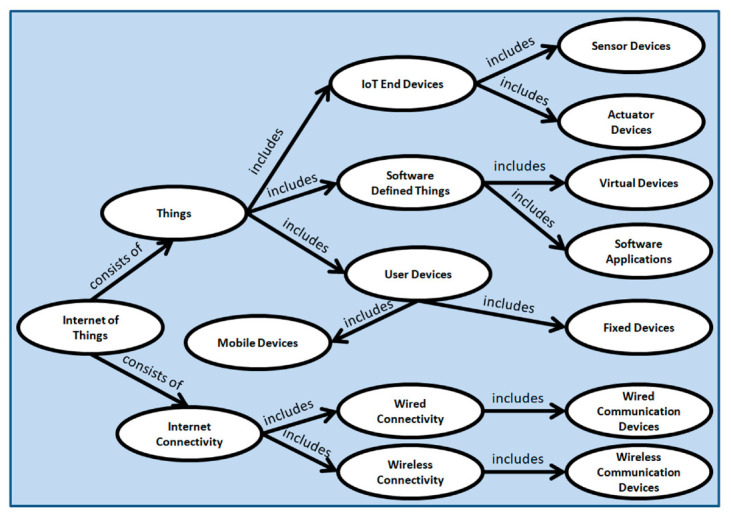
Ontology of IoT visualized as a semantic network.

The internet of things (IoT) paradigm is forwarding towards large scale implementations of hardware and software. It covers a huge number of devices, their connectivity, and related applications which leads to heterogeneity problems within the networks, applications, and connected IoT devices. Semantic technology and ontology-based representation of any large-scale system create a common vocabulary, knowledge, and understanding for different people working within the domain. Ontological representation also helps the building of an abstract system model with the vocabularies of entities and with their functionalities [50]. We have presented the ontology of IoT as a network semantic in Figure 4. The IoT consists of things and internet connectivity. Things include IoT end devices, user devices, and software-defined things. The user devices can be fixed or mobile. The IoT end devices can be sensors and actuators. Internet connectivity can be wired or wireless which consists of wired communication devices and wireless communication devices respectively. The things (sensors, actuators, user mobile devices, software-defined devices) connect via wireless communication devices. The user’s fixed devices connect via wired communication devices.

**Figure 5:**
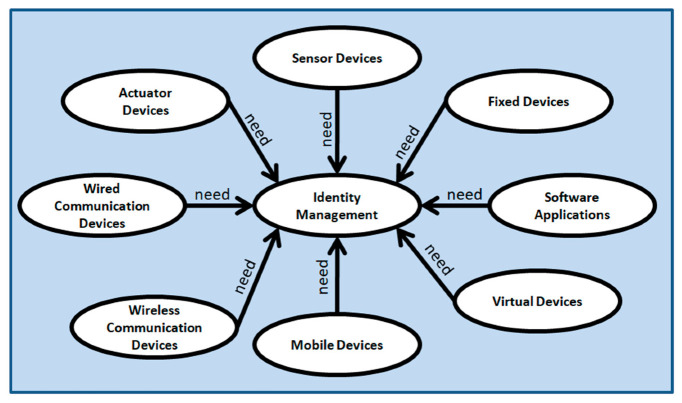
Identity management of IoT entities visualized as a semantic network.

All IoT devices required identity management while connecting to the internet. In Figure 5, we have presented another network semantic considering identity management as the central component. The devices in Figure 5 are considered from the ontology diagram presented in Figure 4. All devices should have identities and these devices need to be authenticated before transferring data between each other.

**Figure 6:**
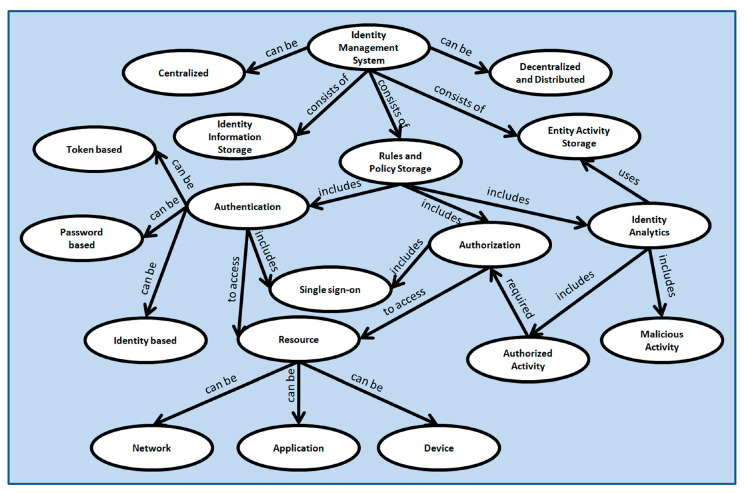
IoT identity management taxonomy visualized as a semantic network.

IoT Identity management taxonomy is presented in Figure 6. Any identity management system consists of different data storage to store identity cardinals of different entities, a set of rules and policies to handle the cardinals and logs of activities performed by the entities. An entity can be a user, a software system, or a hardware device (Figure 5 represents different IoT entities). An identity management system can be centralized or decentralized and distributed. In a centralized identity management system device authentication and authorization are performed by the centralized identity management server and identity information is stored at only one place which is mostly the cloud repository. On the other hand, in a decentralized and distributed identity management model identity cardinal are distributed at different systems and authentication and authorization are performed locally which reduces the network traffic and improves efficiency for real-time systems. Additionally, for large scale deployment of IoT devices and applications centralized identity management systems are not very efficient due to high latency and huge bandwidth requirements [26,51]. The rules and policies include authentication rules, authorization information, and rules and policies for identity analytics. Authentication can be token-based, password-based, and identity-based. Password-based authentication is mostly applicable for user authentication where human interaction is involved. Token-based authentication is mostly used for accessing application programming interfaces (APIs).

Identity-based authentication is more appropriate for a machine to machine communication scenario. In some scenarios, Single sign-on is used where web-based login is involved. A user or system gets access to several systems connected with a single identity management system. A single authentication is performed by the identity management system and the user or system gets access to several systems based on the authorization rules applied. Authentication and authorization of any system or user are performed to allow access to one or several resources. Resources can be a network, an application, or a device. The identity analytics rules use the access logs in the entity activity storage for the detection of malicious activities within the network or system. Activity performed by authorized users or systems is also detected by the identity analytics system. Due to IoT devices and applications’ heterogeneity, activity logs can be very useful for IoT security. Activity logs analysis based on machine learning algorithms and artificial intelligence can enhance IoT security (not discussed in this paper). We will consider it in our future research work. The next section represents our proposed identity management and secure communication model in detail.

### 4.2. IoT Identity Management and Secure Communication Model

As we have presented in the identity management ontology section above, identity management is a complex process that not only includes the cardinal information, but also authentication, authorization, and activity logs. Additionally, due to the heterogeneity in IoT entities, the authentication process can be different based on the context and entities where the authentication is applicable. In this paper, we have only focused on the identity management of IoT devices at the edge-fog layer and secure communication between IoT devices and edge IoT gateways. The identity providers are distributed locally in our proposed model and collaborate via the global identity provider at the cloud layer. The cloud identity provider servers provide scalability and large storage of data which can support the local identity providers as needed. So, one of the main advantages of this model is distributed and decentralized identity management at the edge-fog layer with scalability via the cloud layer for the large-scale deployment of the IoT paradigm.

To avoid confusion, this section starts with the notation of different terms used in this paper followed by all assumptions used in our proposed model, a network model with details on secure IoT communication at the edge layer, detailed operations of each of the components, and ends with session key initialization protocol for secure communication between IoTD and EIoTG.

#### 4.2.1. Notation of Terms

In this paper, we have considered different notations for different terms used within the rest of the paper. The notations of different terms are given in Table 1. The notations of the terms include abbreviate notations of the components, identities of the components as well as different terms used for data encryptions and decryptions at different components to ensure secure communication between those components.

#### 4.2.2. Assumptions

We have considered some practical assumptions in this paper. The assumptions are the preconditions for the implementation of our proposed model. As previously mentioned, there are four components of our proposed model: IoT device (IoTD), edge IoT gateway (EIoTG), the local identity provider (LIdP), and global Identity Provider (LIdP). All these components should have their own local trusted storage (LTS) which should be only accessible from the local trusted applications (LTA). We have added the prefix local to the LTS and LTA to ensure that the trusted storage is within the hardware. The trusted applications are the applications run locally and not remotely executable from other entities within the internetwork. The concept of LTS and LTA is build based on the concept of the trusted execution environment (TEE) [52]. A TEE is an area within the hardware where only trusted entities and trusted applications get access [52,53,54]. As the IoTD, EIoTG, LIdP, and GIdP need to store different security keys, secure and trusted storage is essential for the isolation of those from all external entities [52,53]. The communication of external entities with the trusted environment is not possible and external entities only get access to the limited amount of data shared by the trusted applications (TA) [54]. During the initialization of the system, the IoTD and the EIoTG are registered with the LIdP. The IoTD needs to know which EIoTG it should collaborate with first. The registration includes the registration of hardware addresses of IoTD and EIoTG in LIdP identity registry, EIoTG identity (ID_EIoTG_), and the shared key (SrK_IoTD-EIoTG_) in IoTD’s identity registry. After the manual registration of information in IoTD’s local trusted storage (LTS) and in LIdP’s LTS, the LIdP automatically updated the EIoTG about the new IoTD. The secure messages flow between LIdP and EIoTG is described in Section 4.4. Considering the above-mentioned assumptions and terms notations in Table 1 we have presented the network model of our proposed system below.

#### 4.2.3. Decentralized and Distributed IoT Network Model

**Figure 7:**
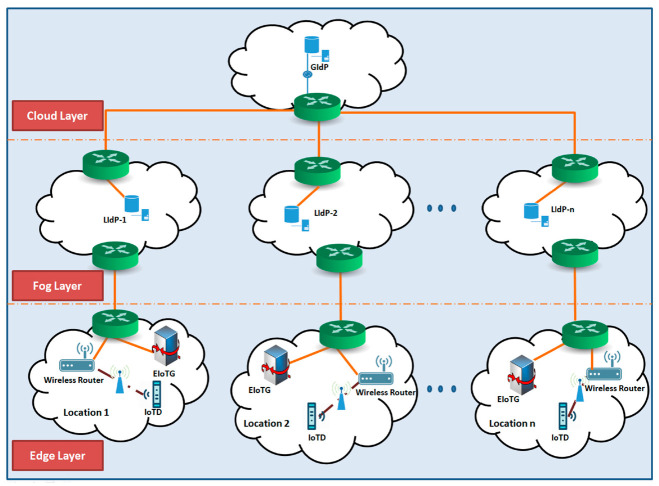
Proposed distributed and decentralized IoT identity management model.

A generalized network diagram of our proposed model is presented in Figure 7. In this model we have suggested identity providers (IdP) at fog and cloud layers. The fog layer IdPs work as local identity provider (LIdP) and the cloud layer IdPs work as global identity provider (GIdP). Identity providers (IdP) work as a trusted third-party entity within the IoT infrastructure. The main task of IdPs is to store identity information edge IoT gateway (EIoDG) and end IoT devices (IoTD) like sensors and actuators which are mostly involved for some specific tasks. The GIdP servers only collaborate with the LIdP servers, with other GIdP servers, and IoTDs when it moves between EIoTGs. But the LIdP servers communicate with the EIoTG and IoTD as well as GIdP as per requirement. As the proposed model localizes the identity solution near to the end devices, it reduces the network traffic as well as increases the efficiency of the network. One of the biggest advantages of our proposed decentralized and distributed identity management model is the isolation of networks. An isolated decentralized and distributed identity management model will allow efficient accommodation of large numbers of IoT devices which is under deployment all over the world. The identity management servers not only assign identity for new entities within a location but also store and shares keys and works as a key management server. The keys shared by the identity management servers are further used for encryption and decryption of data at different IoT devices, gateways to ensure secure communication. A secure communication channel is essential to ensure different network-level attacks.

**Figure 8:**
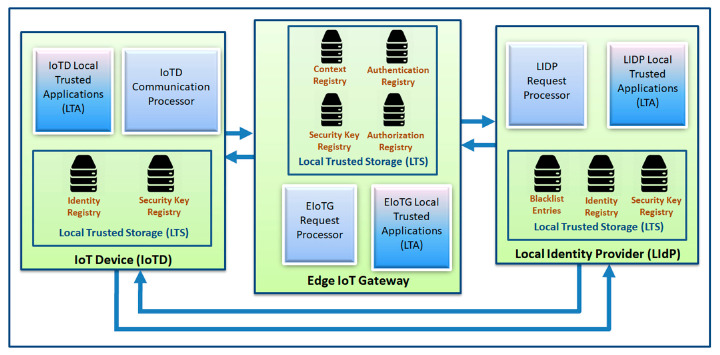
Internal architecture of security communication modules in IoTD, EIoTG and LIdP.

**Figure 9:**
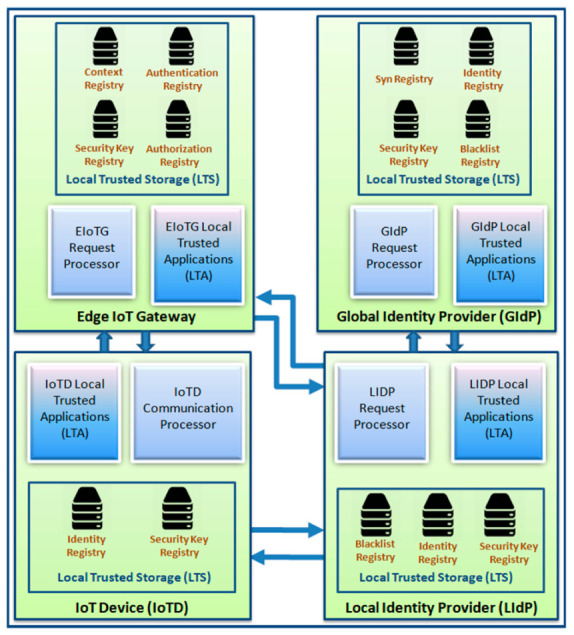
Internal architecture of security communication modules in IoTD, EIoTG, LIdP, and GIdP.

A detailed block diagram with the internal architecture of secure communication modules of different components of our model is presented in Figure 8 and Figure 9. There are three components in Figure 8, the end IoT device (IoTD), the edge IoT gateway (EIoTG), and the local identity provider (LIdP). The IoTD and EIoTG are located at the edge layer and the LIdP should be placed at the fog layer of an IoT network. Our proposed security modules can be implemented within the above mentioned IoT network components (IoTD, EIoTG, LIdP, and GIdP) to ensure the secure device to device communication as well as to secure different security cardinals at different devices. We called this a secure communication module because it will ensure a secure device to device communication. Different parts of our proposed security modules inside each of the components are described below.

There are three main parts inside the IoTD: communication processor, local trusted storage (LTS) and local trusted applications (LTA). The communication processor handles all external communications. The LTS has two registries: identity registry and security key registry. The identity registry is the repository for IoTD’s own identity, the identity of the EIoTG where the IoTD is currently connected, and the identity of the previously connected EIoTG in case the IoTD has been moved from another EIoTG. The IoTD may need to store the identity of the LIdP as well when it is shared by the EIoTG. But the LIdP’s identity is stored in IoTD for a certain time and removed automatically, as the IoTD’s are resource-constrained devices. The security key registry holds the security keys which are used for secure communication of the IoTD with the EIoTG and the LIdP. The LTS is only accessible via the LTA and no direct access from the communication processor is allowed.

There are also three main parts inside the EIoTG: request processor, LTS and LTA. The request processor handles all communication requests with the connected IoTD and LIdP. The LTS in the EIoTG has four parts: security key registry, authentication registry, authorization registry, and context registry. The security key registry holds the security keys for secure data communication to and from external devices (IoTDs and LIdP). The authentication registry holds information related to authentication of connected IoTDs which has been assigned by the LIdP server. The authorization registry holds the authorization information of the connected IoTD. Each EIoTG maintains individual authentication and authorization registry which allow quick authentication and reduce network traffics compared to a centralized authentication and authorization system. The context registry holds different context information about the connected IoTD. All the registries with the LTS use the IoTD_ID_ as a primary key. The LTS inside EIoTG is also only accessible via LTAs. External interaction with the registries inside the LTS is not allowed. Like the EIoTG, the secure communication modules inside the LIdP also consist of three parts: request processor, LTS and LTA. The request processor handles all communication requests with the connected IoTD and EIoTG and other LIdPs within the same location and GIdP in which it is connected. The LTS inside LIdP consists of a security key registry, identity registry, and blacklist registry. The security key registry and identity registry are quite similar to the registries inside IoTD and EIoTG. The new registry inside LTS of LIdP is the blacklist registry that stores information about blacklisted devices which may have been compromised. The blacklist registry is filled from the data collected from the intelligent EIoTG which analyzes unexpected interaction of IoTD with the EIoTG based on their context and reports incident to the LIdP it is connected.

Figure 9 is an extension of Figure 8 where in addition to the internal architectures of IoTD, EIoTG, and LIdP, we have added the internal architecture of GIdP as well. The LIdPs and GIdPs have almost similar internal architecture but the communication patterns are different. The GIdP is only responsible for sharing identity information with LIdPs and with other GIdPs. The only architectural difference between a LIdP and a GIdP is GIdP has an extra registry called Sync registry where it keeps the track of time to time synchronization with LIdPs and other GIdPs. To simplify the diagram, we have only shown a single LIdP and a single GIdP in Figure 9 but, it should be more in reality. The next section represents the primitive state machine approach for identity management and authentication processes in our proposed model.

### 4.3. Primitive State Machine Representation of IoT Identity Management

**Figure 10:**
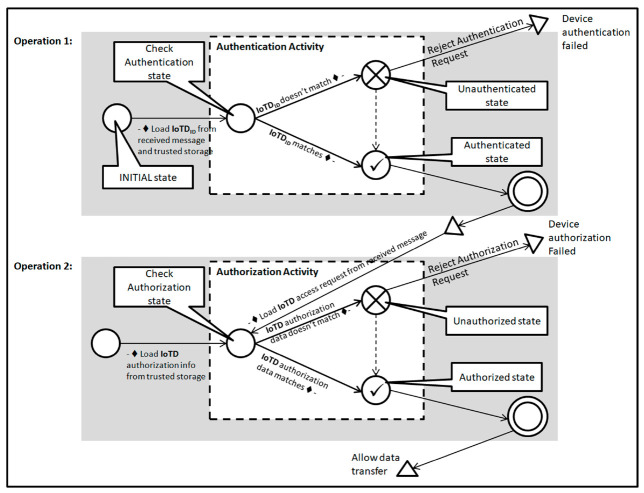
Authentication and authorization for IoT device (IoTD) at edge IoT gateway (EIoTG).

In this section, we have represented primitive finite state machines (pFSM) of different operations performed by our proposed system. In Figure 10, we have represented a primitive FSM diagram for IoT device authentication and authorization operation performed at IoT Gateway. A pFSM [55], is a simple way of representation of any complex system. Any complex system with several operations and activities can be represented by dividing the operation and activities separately by different states and activity blocks. In a pFSM representation, an operation can consist of one or multiple activities. The results from one activity can be used as an input for the next activity within the same operation. The result from one operation can be used as input to the next operation or as a termination of the execution. We have two operations in Figure 10. The first operation has an activity namely authentication activity. The second operation has an activity called authorization activity.

At first, the IoTD_ID_ is loaded from the request message from the IoT device and the trusted storage within the EIoTG. After the verification of the identity at the check authentication state a decision is made whether the device is authenticated or unauthenticated. If the IoT device is proved as authenticated, then the next operation is the authorization. The authorization is required as the IoT devices can access different data storage and may need to perform read or write operations at different tables. At the authorization activity within the second operation, the access request is loaded from the message received from the IoT device and the authorization table for that specific IoT device is loaded from the trusted storage in the IoT gateway. After the verification of authorization, data read from the message and write operation to the storage is performed if it is a message for reading data from an IoT device. In case it is a read data from the gateway, data is read from the IoT gateway storage after authorization and an encrypted message is sent to the IoT device.

**Figure 11:**
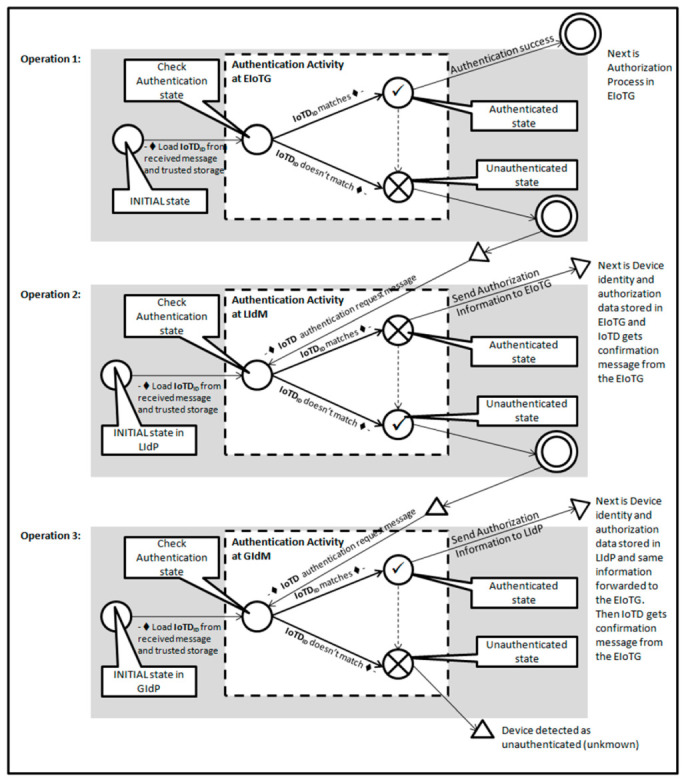
Authentication process for an IoTD at EIoTG, LIdP and GIdP.

There could be scenarios where IoTD is not authenticated at the EIoTG as it has been moved from another EIoTG. A pFSM for this situation is presented in Figure 11. At first, the EIoTG checks the identity of the IoTD, and if the device is authenticated it proceeds with authorization as described above. In case the EIoTG can’t find any information about the IoTD in its’ storage, it sends an authentication request to the LIdP for verification of the identity of that IoTD. In case the IoTD was previously connected to an EIoTG which is within the range of that specific LIdP. It checks the identity of that IoTD and sends authorization information to the EIoTG.

In case the IoTD was not previously connected to an EIoTG within the range of that LIdP, it forwards the request to the GIdP. The GIdP checks the identity of the IoTD as it is synchronized with the LIdPs. In case the GIdP finds an entry for that specific IoTD, it confirms that identity and sends authorization information to request the LIdP. The LIdP saves the identity and authorization information in its own storage and forwards the same information to the EIoTG. The EIoTG saves the information in its own storage and confirms the IoTD.

### 4.4. Secure Communication Protocol for IoT

To ensure secure communication between different components of IoT paradigm, we have designed a new secure communication protocol. The main communicating parties are IoTD and EIoTG. Our proposed secure communication is initiated with session key initialization between IoTD and EIoTG. The key agreement protocol is designed based on the elliptic curve Diffie-Hellman ECDH [56]. ECDH is a key agreement protocol that is designed based on elliptic curve cryptography (ECC). ECC is a lightweight public-key cryptography algorithm that is popular for ist’ small key size and less computational resource requirement compared to other public-key cryptographic algorithms [57]. We have considered ECC because IoT devices are resource-constrained. After the session key establishment, secure data communication between IoTD and EIoTG is performed using symmetric key cryptography [58,59], which have a nearly similar computational requirement and not much dependent on key size [59].

**Figure 12:**
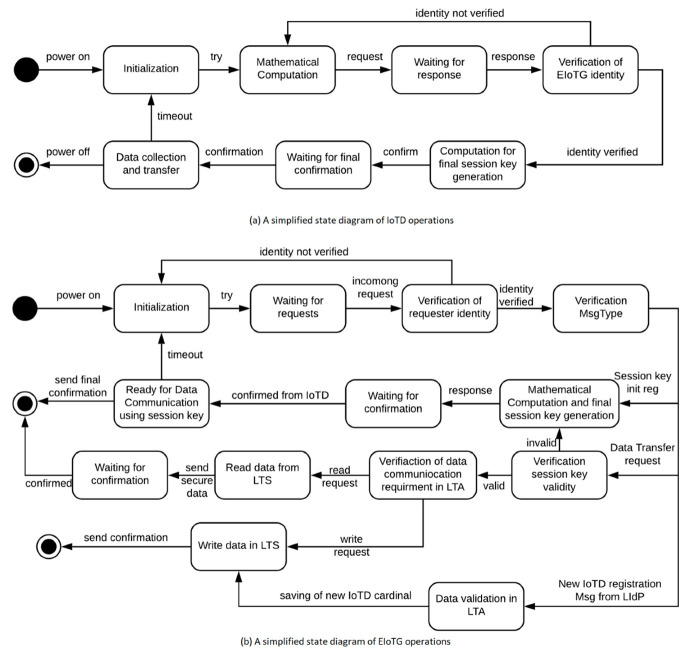
State diagrams of operations of IoTD and EIoTG.

To describe the operation of our secure communication protocol, we have presented a simplified state representation of IoTD operations in Figure 12a and a simplified state representation of EIoTG operations in Figure 12b. In Figure 12a the IoTD initiated after the power on, it performs mathematical operations and make a request for the session key initialization to the EIoTG. After the request, the IoTD waits for the response from the EIoTG. After getting a response from the EIoTG, IoTD verifies the identity of the sender and if the identity is valid, it computes the final session key. After session key generation it sends a confirmation to the EIoTG and waits for the final confirmation from the EIoTG. If it gets a valid confirmation the next state is the data collection and transfer with the EIoTG. If the identity of the EIoTG is invalid the device goes to the mathematical calculation state and tries to re-send the request. The operation of EIoTG is initiated after the power on (Figure 12b). After the initialization, the EIoTG waits for request from IoTDs or LIdP. If it gets a request, it verifies the identity of the request in the next state. If the identity is invalid it goes to the initial state. If the identity is verified, the next state is the verification of message type. There are three types of messages handled by the EIoTG: session key initialization message from the IoTD, data transfer request from IoTD, and registration of new IoTD message from the LIdP. If the message type is session key initialization, the EIoTG performs a mathematical calculation, generates the final session key, and sends a response to the IoTD. After sending the response it waits for the confirmation from the IoTD. If the IoTD confirms, it sends the closing final message to the IoTD and that specific operation ends. If the message type is data transfer request from a IoTD, it validates the session keys and if the session key is valid, it verifies if it is a read or write request. If it is read request EIoTG stores data. If it is a read request it reads the data from storage sends to the IoTD and the read/write operation also ends here. In case the message type is a registration of new IoTD. The EIoTG validates the data (cardinal information) sent from the LIdP and stores in local storage. After storing the cardinals of the IoTD to its own storage it sends a confirmation to the IoTD and the operation ends. Detailed message flows between IoTD and EIoTG for the session key establishment in our proposed model is presented below.

**Figure 13:**
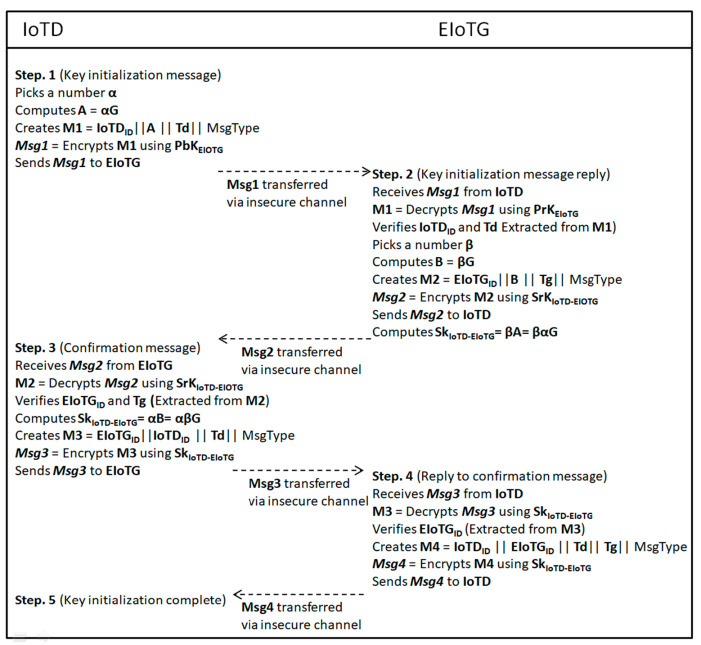
Message flow diagram for session key initialization between IoTD and EIoTG.

In our proposed model IoTD and EIoTG communicate over an insecure channel. To perform secure communication over insecure channels, we have proposed symmetric key cryptography based encryption and decryption. But for the implementation of symmetric-key cryptography, the session key establishment is essential [59]. Figure 13 represents the message flows between the IoTD and EIoTG for the establishment of the session key. In the next section, we have presented the simulation and performance measurement results of our proposed model using different use case scenarios.

## 5. Results and Performance Evaluation

In this section, we present the conceptual validation and performance evaluation of our proposed model with a concise and precise description of our experimental results. The validation and performance measurements are performed based on three different scenarios. We have used two different simulation tools. Scyther is used for formal security analysis. SPIN model checker is used for the proof of concept. The rest of Section 5 is arranged as follows: in Section 5.1 we have represented the performance evaluation of our proposed identity management model. In Section 5.2, we present the security analysis of the proposed secure communication protocol considering different threats for IoT paradigm as well as considering the basic security requirements for IoT systems followed by verification using the Scyther security verification tool in Section 5.3. Section 5.4 represents the validation of our proposed model using the SPIN model checker tool.

### 5.1. Identity Management Model Performance

It is very crucial to evaluate the performance of any proposed identity management (IdM) solution/model because IdM is the first step toward data security in any digital/software system [60]. The identity management process is also the first step toward ensuring the privacy of data. Though in this paper, we are more concerned about identity management of IoT devices and not considering the user identity management, it is still passively connected to the user data. Because by the end of the day, most of the IoT devices are deployed in human society and involved in processing data collected from the users and their surroundings. In this section, we have presented the evaluation of our proposed IoT device identity management system. We have analyzed the performance of the proposed IdM based on nine main requirements identified by Boujezza et al. [61] as well as two more evaluation criteria discussed in [62].

#### 5.1.1. Security

Though device identity management is the first step towards security, the identity management system itself needs to be secure enough to protect the cardinal information it stores and processes [60]. As described in Section 5.3, we have verified our proposed identity management model using the Scyther security verification tool. Our proposed model will be able to provide network security by ensuring secure communication between different components. Additionally, the system will be able to provide device-level security using the trusted storage module and trusted application modules.

#### 5.1.2. Privacy

As identity management, authentication, and authorization processes work together [61] as doorkeeper for access to data and information processed by any IoT device, user cardinals, and data privacy management capability of an IdM system is very crucial. We have designed the internal architectures (Figure 3, Figure 8 and Figure 9) of different components in considering the privacy of data. Though privacy management is out of the scope of this article and will be elaborated in our future papers, still we have designed our system considering privacy management at the edge/fog gateway of the paradigm. The data privacy in our model will be handled by the decision support module (Figure 3).

#### 5.1.3. Trustworthiness

A system becomes trustworthy based on its functionality and performance. Our proposed model is trustworthy as we have verified the security performance of our model using a popular security verification tool. Additionally as we have described above, our proposed model will be able to handle data privacy at the edge layer and will protect sensitive information during its transfer over insecure channels.

#### 5.1.4. Mobility

The mobility handling capability of IdM systems is essential because the IoT nodes are more mobile and not static. Our proposed identity management system will ensure the mobility of the devices. When a device moves from one location to another, it gets registered with the edge gateway with few easy automatic steps performed by the system without human interactions.

#### 5.1.5. Usability

The usability of any identity management system is crucial because people prefer the systems which are easy to use and easy to configure [61]. In our case, the proposed model doesn’t need many configurations by the users. It will perform all the tasks itself after the initial configuration. The system can be able to automatically provide alarms via SMS or emails based on the threats it detects and in case a connected IoT device is compromised. Additionally, as described above, device mobility is handled by the system, and users do not need to reconfigure the system when an IoT node moves to another location.

#### 5.1.6. Affordability

The affordability of any system is important for the wide use of any system. Our proposed identity management model will be affordable in the sense that it is designed based on lightweight security protocols and doesn’t need expensive hardware. Additionally, we have proposed GIdP in our model which can be implemented using cloud computing and doesn’t need to be deployed locally by individual organizations.

#### 5.1.7. Law enforcement

Our proposed model can be extended with law enforcement at the edge gateways. These can be done within the decision support module presented in Figure 3. The system will be able to provide automatic decision support based on the rules implemented on it.

#### 5.1.8. Interoperability

The proposed system can be extended with the interoperability capability while the IdPs collaborate. Our proposed identity management model is generic and it can be implemented within any use case scenario. So, in a sense, it can be used as an independent service for identity management of different types of IoT solutions. The main advantage here is we don’t need much information while configuring the devices for the first time as a result it is adaptable to any use cases.

#### 5.1.9. Functionality

The proposed system has several functionalities: authentication, authorization, secure key management, context management, automated decision support (as presented in Figure 3, Figure 8 and Figure 9). The system will handle module based concepts to provide different functionalities. This means the internal components will be able to alone process data within individual modules.

#### 5.1.10. Scalability

Another evaluation criteria for any identity management system is scalability [60]. Our proposed system provides scalability via the cloud layer global identity provider. As we have proposed a distributed identity management model for edge IoT devices, it will be possible to accommodate a huge number of IoT device deployment.

#### 5.1.11. Administration

Our proposed system has easy administration. The proposed system can be administered using a simple web interface or terminal-based access to the system. The administration should be easy because we have suggested a one-time configuration at the initial deployment stage, after the initial configuration the device activity can be monitored via a web interface or terminal. We haven’t discussed the details of the web interface in this paper, but it is possible to improve our proposed system with a web interface. In case a web interface is used with this model, we will suggest before configuring the web access to the administration page of our proposed system, secure web access must be ensured.

#### 5.1.12. Comparative Analysis

Based on the above discussions, in Table 2, we have presented a comparative analysis of different identity management solutions with our proposed model. These IdM solutions are also compared with our approach in the related works section of this paper. The results show that most of the designers of IoT identity management systems didn’t address all the above-mentioned criteria during the design of their solutions. None of the papers discussed affordability and law enforcement in their proposed identity management solution.

### 5.2. Informal Security Analysis

In this section, we have presented the informal security analysis of our proposed model and proposed protocols. Different security aspects of our proposed model are evaluated concerning common security requirements as well as concerning different types of security attackers. The common Security requirements include mutual authentication, confidentiality, integrity, and availability. The attacks include replay attack, denial of service (DoS) attack, man-in-middle attack, physical attack, and impersonation attack [63].

#### 5.2.1. Mutual Authentication

In our proposed model the IoTD and EIoTG share their identity via secure communication messages and verifies their identity mutually before performing any data transfer. Additionally, the communication between EIoTG(s), LIdP(s), and GIdP(s) are done via secure communication and each entity are mutually authenticated before the actual data transfer between them.

#### 5.2.2. Confidentiality

In our proposed model different keys as well as data confidentiality is ensured. The session keys are used for encryption and decryption of messages at the sender and receiver ends respectively. In our proposed model the authenticated devices only share the session keys between them using ECDH protocol. The intruder is not capable to retrieve the session keys. Additionally, all the cardinals are stored in the trusted storage and only accessible via the trusted applications. Any physical tampering with these devices reset the device to its’ initial configuration and the device data can be erased based on the severity of the attacker’s malicious activity. Data confidentiality is ensured by secure data communication between authenticated devices only.

#### 5.2.3. Integrity

Integrity ensures the tempering with the messages on the way between the sender and the receiver. To break the integrity an attacker needs to know the secret session key used to encrypt the message. As the secret session keys are transferred over the secure communication between two authenticated entities before the data transfer process, it is not possible to capture the secret session key and to eavesdrop on the messages transferred between two network entities in our proposed model.

#### 5.2.4. Availability

The availability is ensured by localization of services and reducing long-distance network traffic and complex authentication from a centralized cloud server. In our proposed model, the EIoTG and IoTD authenticate each other and reduce the interaction with a centralized cloud server or authentication via a cloud server.

#### 5.2.5. Reply Attack

Most of the time devices communicate over insecure channels. In case an authentication takes place before the setup of a secure data transmission channel, attackers may eavesdrop on the network traffic and may replay to the sender later presenting themselves as the trusted network entity. This effects further communication and attackers can collect all the data by presenting themselves as an authenticated entity. In our proposed model, a replay attack is protected by the initial registration process. All the components in our proposed model make secure communication using fresh random values and attackers can not reuse the old random values even they eavesdrop to any message. Additionally, we have proposed a model where context information (time, location) is crucial. It will be easy to detect malicious behavior based on the context information shared by an attacker.

#### 5.2.6. Denial of Service (DoS) Attack

In our proposed model EIoTG and IoTD only communicate with the authenticated and trusted predefined entities. The communication messages consist of different context parameters timestamps, location information. If an attacker sends an invalid request, the system will be able to detect the malicious attitude and will block that entity and will ignore all the packets sends from that specific entity.

#### 5.2.7. Man-In-The-Middle Attack

In our proposed model, the authentication process takes place within secure communication. The devices start initial handshaking with secure messages using ECDH based session key initialization and after the key initialization, the data transfer is performed. As a result, man-in-middle attacks will not be possible. The session keys are valid for a certain time and are further exchanged using a fresh nonce. So, even if the attackers compute a session key, he/she will not be able to use it at the next step.

#### 5.2.8. Physical Attack

Sometimes devices may be physically compromised. In those situations, cardinals of other entities within the network are protected by the trusted storage. Any kind of malicious access to these trusted storage is not allowed and devices would be able to go to its’ initial setup to avoid the physical attacks.

#### 5.2.9. Impersonation Attack

Our proposed model is resistant of impersonation attack because in an impersonation attack a malicious node masquerades as a trusted valid node. It is not possible in our proposed model because to indicate as a trusted node the malicious node needs to have all the cardinals initially stored in the trusted storage of a real valid device, which is not possible.

#### 5.2.10. Forward and Backward Secrecy

In our proposed model session keys are generated using fresh random numbers and are not dependent on each other. So, it is not possible to generate or reuse any session keys from the past and not guess the previously generated session keys using future session keys. So, our proposed model ensures forward and backward secrecy.

### 5.3. Formal Security Analysis

In this section, we have presented the formal security analysis and security performance measurement of our proposed model and proposed protocols. We have verified the security performances of our proposed model and related protocols using the Scyther tool [64] v1.1.3. The simulation was run in an Intel (R) Core (™) i7-6500 CPU @ 2.50 GHz 2.59 GHz and 16.0 GB internal memory (RAM) with Windows 10 Professional operating system also in Intel (R) Core (™) i3-CPU M @ 2.40 GHz and 8.0 GB internal memory (RAM) with 64-bit Ubuntu 16.04 LTS operating system. The results from both systems are the same. Scyther is a popular tool for automated verification of security protocols, which was introduced in the year 2008 [65]. It has been used in many recent security research [66,67,68,69,70,71]. Scyther is a Python based tool [65,67] which is available for Linux, Windows, and Mac OS X environments [64,65].

#### 5.3.1. Scenario Description

**Figure 14:**
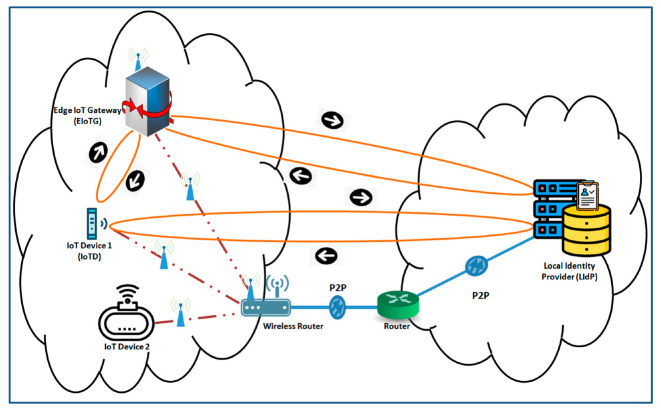
Identity management via LIdM when IoTD and EIoTG are at same network.

We have considered two different scenarios. In these scenarios, the IoTD and EIoTG are located at the edge layer, the LIdP is located at the fog layer, and the GIdP is located at the cloud layer. The first scenario is presented in Figure 14. In this scenario, an IoTD joins to an EIoTG for the first time. As described in Section 4.2.2, before the first time joining the IoTD is registered with the LIdP. The EIoTG and IoTD are considered within the same network (edge) and the LIdP server is placed within another network (fog). For simplicity, we have not considered the cloud layer in Figure 14.

**Figure 15:**
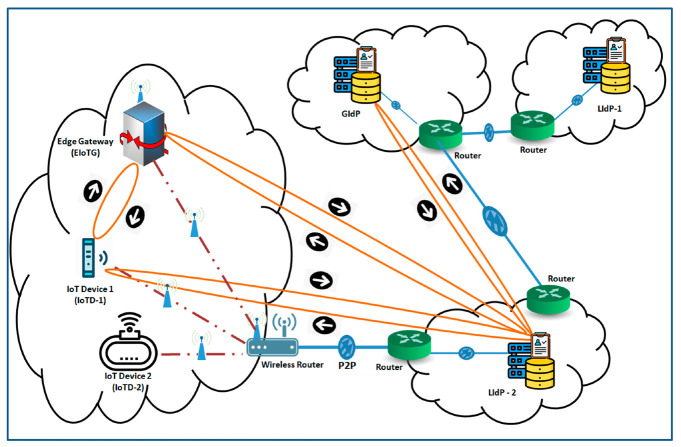
Identity management via LIdP and GIdM when IoTD and IoTG are at same network.

As the IoT devices may have mobility, IoTD might leave an EIoTG and join a new EIoTG. Identity verification is required in a scenario where an IoTD leaves an EIoTG and join another EIoTG. For this kind of scenario, a LIdP is not enough to verify the identity of IoTD, so GIdP is required. Figure 15 presents a scenario with LIdP-1, GIdP and LIdP-2.

In the scenario in Figure 15, an IoTD-1 was previously attached with LIdP-1 at location 1 and now it has moved to a new location (location-2) and requested to join the EIoTG at the new location. As the IoTD-1 is for the first time joining this new EIoTG, it has no identifying information for IoTD-1 and will need to verify the identity via the LIdP-2 at location 2. However, as this IoTD-1 is not registered with the LIdP-2, the LIdP-2 sends a request to the GIdP to verify the identity of the IoTD-1. The GIdP continuously synchronizes its registry with the nearest LIdPs. So, it checks its registry and confirms the identity of the IoTD-1 to the LIdP-2. The LIdP-2 returns to the EIoTG at location 2 with the identity information of the IoTD-1.

#### 5.3.2. Scyther Simulation Results

In this section we have presented the Scyther verification results connected to the above-mentioned scenarios. For verifications of security properties of any protocol using Scyther tool, the models and related different security claims: Secret, Alive, Weakagree, Commit, Niagree, Nisynch are specified using Security Protocol Description Language (spdl) [65,67]. We have verified that our proposed model and protocols verify all the security claims available in the Scyther tool.

**Figure 16:**
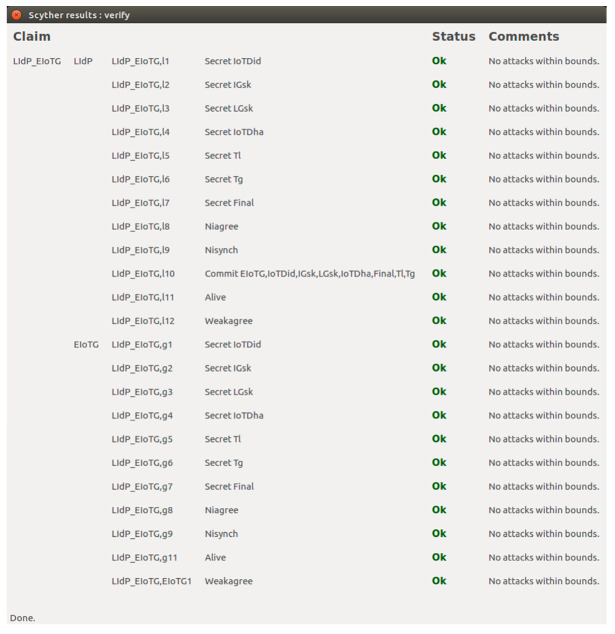
Identity parameters initialization between LIdP and EIoTG.

Figure 16 represents the result of the implementation of the scenario for security cardinal transfer between LIdP and EIoTG using the *spdl* language. As presented earlier, in our model after the initial registration of an IoTD with the LIdP, the LIdP communicates with the EIoTG for the secure transfer of security cardinals of the new IoTD. In Figure 16, the ‘IoTDid’ is the identity of the IoTD, ‘IGsk’ is the shared secret key for IoTD, and EIoTG, LGsk is the session key generated between EIoTG and LIdP. The ‘IOTDha’ is the hardware address of the IoTD, ‘Tl’ is the timestamp shared by the LIdP, ‘Tg’ is the timestamp shared by the EIoTG, and ‘Final’ is the closing message sent by the LIdP to the EIoTG. The Secret claims in the LIdP and EIoTG verify the confidentiality [65,66] of the identity of the EIoTG, IoTD, the hardware address of IoTD, the shared key for IoTD and EIoTG, the session key between EIoTG and LIdP, the timestamp at LIdP and EIoTG and Final message. It means unauthorized access [65,66] to all these parameters are not allowed by the proposed protocol. The Commit claims in Figure 16 verify the commitments on synchronization between LIdP and EIoTG on the above-mentioned parameters.

**Figure 17:**
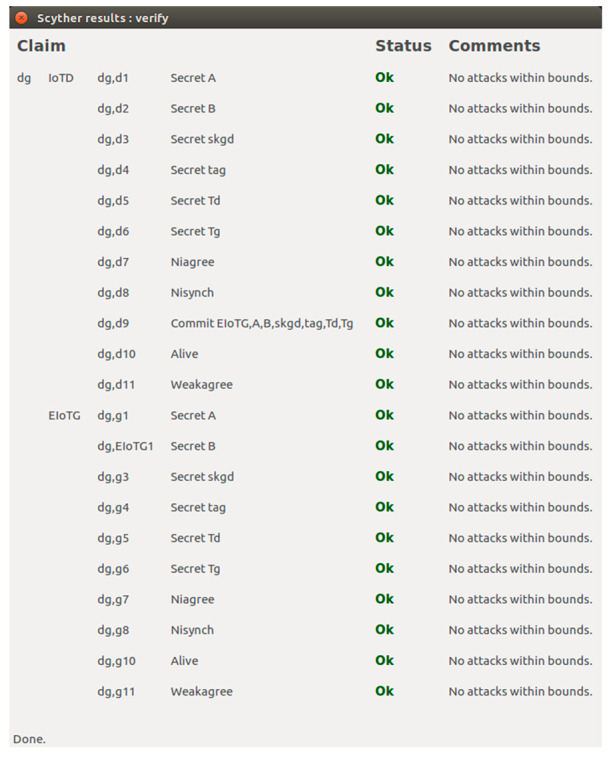
Scyther verification result for session key initialization between IoTD and EIoTG.

Figure 17 represents the result of the implementation of the scenario for session key initialization between IoTD and EIoTG using the *spdl* language. For secure communication, the IoTD and EIoTG generate session key using a combination of ECDH and secret key data transfer between them. In Figure 17, the ‘Tag’ represents the message type, the ‘A’ and ‘B’ are the values generated at the IoTD and EIoTG respectively as described in Figure 13. The ‘Td’ and ‘Tg’ are the timestamps generated at IoTD and EIoTG respectively. The time information is important for detecting malicious activity within the network. The Secret claims in the IoTD and EIoTG verify the confidentiality [65,66] of the identity of the EIoTG, IoTD; the secret parameter ‘A’ (used for ECDH), the secret parameter ‘B’ (used for ECDH), the session key for IoTD and EIoTG, and the timestamp at IoTD and EIoTG. The Commit claims in Figure 17 verify the commitments on synchronization between IoTD and EIoTG on the above-mentioned parameters.

**Figure 18:**
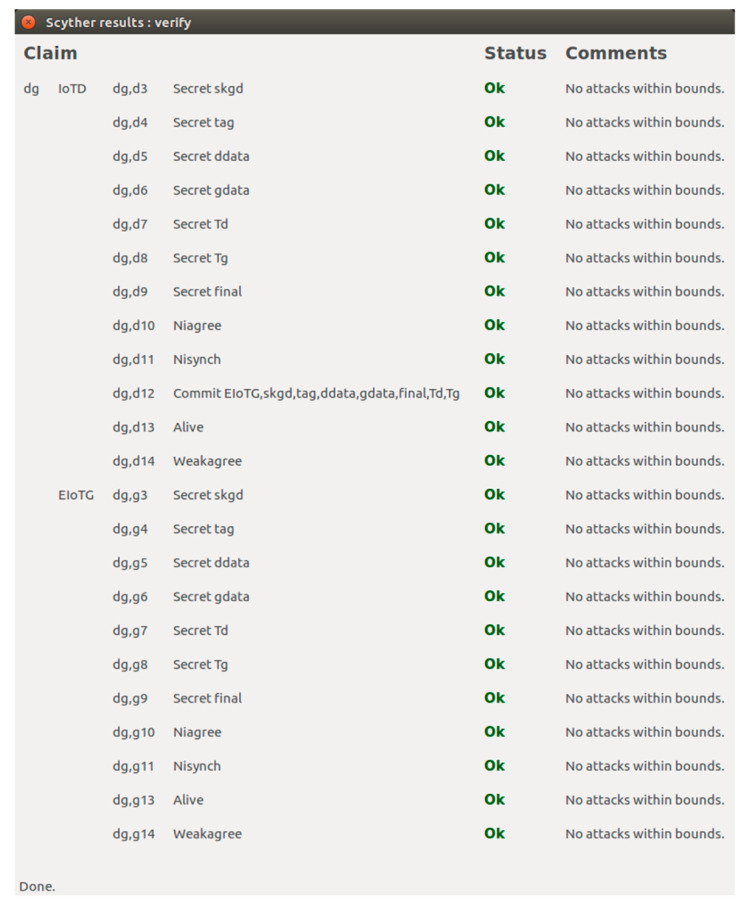
Scyther verification result for secure data transfer between IoTD and EIoTG.

Figure 18 represents the results of the implementation of a secure data communication scenario for IoTD and EIoTG. In this verification, the secrecy of the session key, the secrecy of data at the IoTD, the secrecy of data at the EIoTG, and secrecy of data during transfer between IoTD and EIoTG are proved. In Figure 18, the ‘skgd’ is the session key for data transfer between IoTD and EIoTG, the ‘tag’ is the field for message sequence, the ‘ddata’ is the data at the IoTD, the ‘gdata’ is the data at the EIoTG, the ‘Td’ is the timestamp at IoTD, the ‘Tg’ is the timestamp at EIoTG and the ‘Final’ is the closing message send between IoTD and EIoTG after successful data transfer between them. The Commit verifies the commitments between the IoTD and EIoTG on the identity of the EIoTG, IoTD, the secret of the session key, secret of tag, secret of data at the IoTD, the secret of data at the EIoTG, the secret of timestamp at IoTD, the secret of timestamp at the EIoTG and the secret of the final closing message transferred between IoTD and EIoTG. The Niagree claims in Figure 16, Figure 17 and Figure 18 prove that the messages transferred between the senders and the receivers are unchanged [66]. The Nisynch claims in Figure 16, Figure 17 and Figure 18 prove that the messages transferred between the sender and the receivers in the respective models have reached without any interruption and correct messages are delivered to the receivers [66,68]. The Alive claims in Figure 16, Figure 17 and Figure 18 represent that certain tasks are performed by the specified entities [66,68]. The impersonation attack protection is verified by the Weakagree as claimed in Figure 16, Figure 17 and Figure 18 [66,68].

### 5.4. Verification of Proposed Model

**Figure 19:**
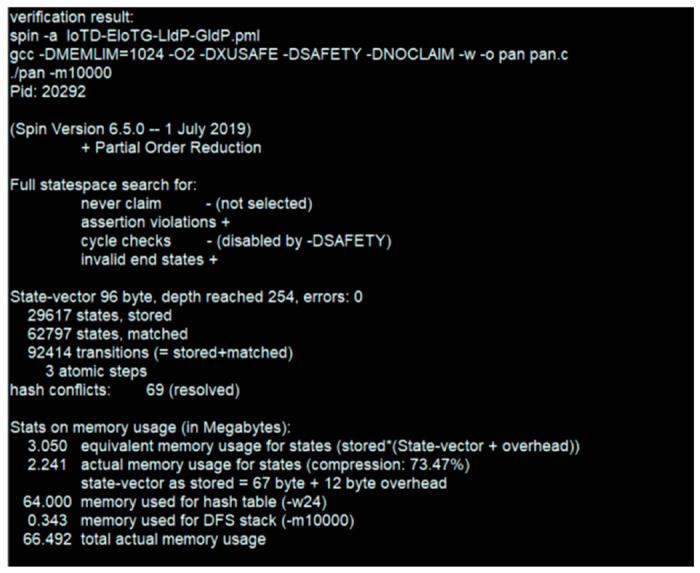
SPIN Verification results for communication between IoTD, EIoTG, LIdP and GIdP.

In this section, we have presented the verification of our proposed model. We have verified the model using the SPIN tool. SPIN is a powerful tool for the simulation and verification of conceptual models. We have used the SPIN tool for the construction of our verification models and checked if the proposed model fulfilled the properties of the proposed system requirements. PROMELA specification language is used for writing the abstract specifications of the models as SPIN allows specifications written using PROMELA [72]. We have used SPIN tools in the basic command-line mode as well as the graphical mode in iSPIN. To reduce the complexity of any model, it is common to avoid the implementation details, and communication protocols [73]. Promela codes consist of three main parts: data types, message channels, and processes [72]. The four components of our model: IoT device (IoTD), edge IoT gateway (EIoTG), the local identity provider (LIdP), and global identity provider (GIdP) are presented as processes. We have presented each network component with different states as *proctype* and different communication activities using the *channel*. Figure 19 represents the SPIN verification results for communication between IoTD, EIoTG, LIdP, and GIdP. It shows that there is no error in our proposed model and the SPIN tool automatically generated total 92,414 states and total actual memory usage is 66.492.

**Figure 20:**
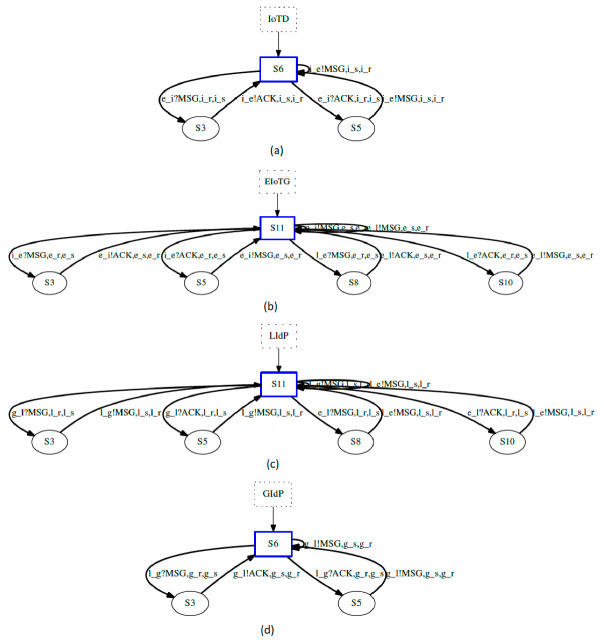
SPIN generated automata views for communication senarios of different components (**a**) IoTD (**b**) EIoTG (**c**) LIdP and (**d**) GIdP.

Figure 20 represents the automata views for communication scenarios of different components in our proposed model. As shown in Figure 20a the state representation of message flow between IoTD and EIoTG, in Figure 20b the state representation of message flow between IoTD and EIoTG, and EIoTG and LIdP, in Figure 20c the state representation of message flow between LIdP and EIoTG, and LIdP and GIdP, and in Figure 20d the state representation of message flow between LIdP and GIdP.

**Figure 21:**
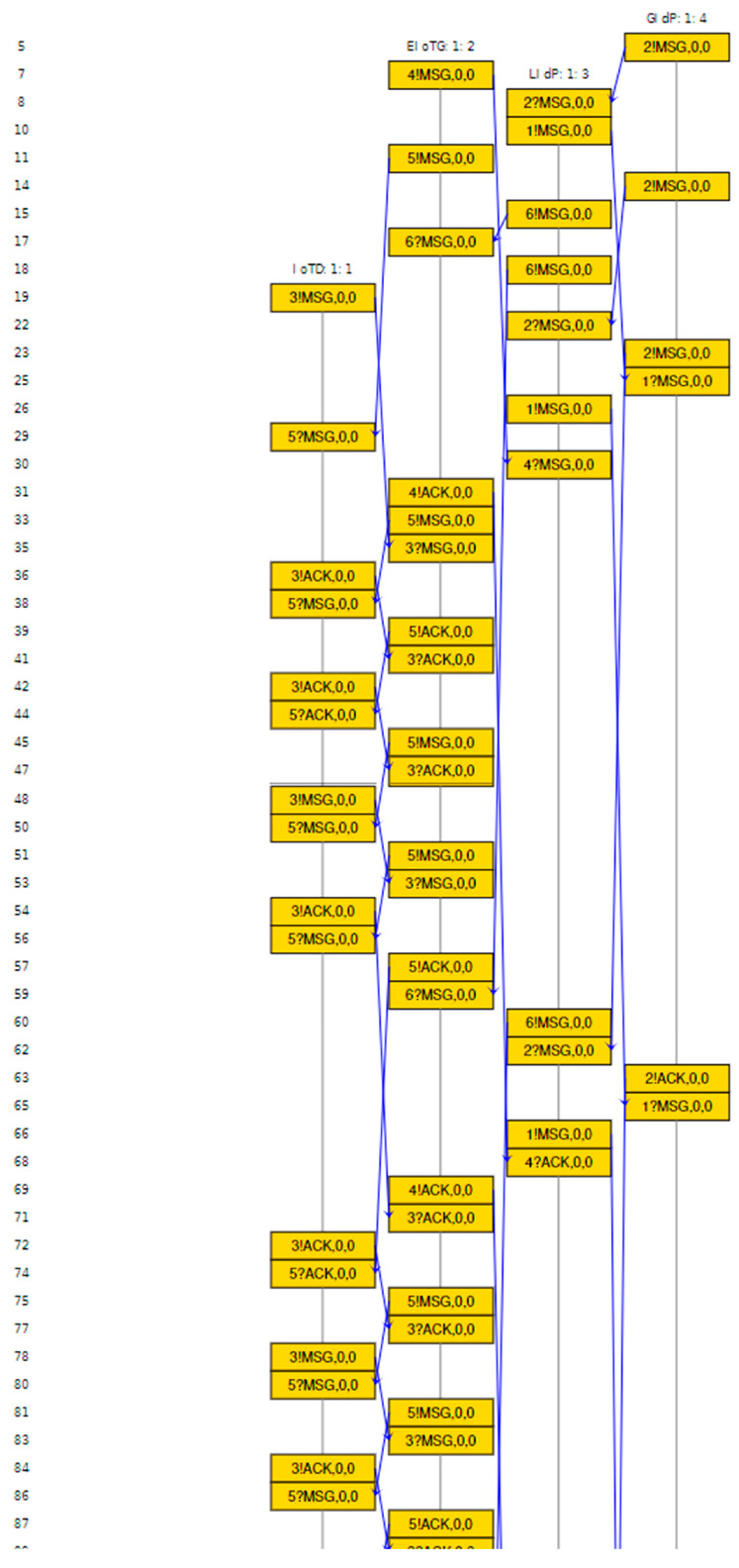
Simulation results for communication between IoTD, EIoTG, LIdP and GIdP using SPIN.

Figure 21 represents the simulation results of our proposed model, here four processes communicate over message channels. There are two different message types generated by each process: MSG and ACK. When a process receives a message of type MSG from another device, it replies to that message with a message type ACK. The devices continuously keep monitoring the channels receiving messages from the other devices and replies with acknowledgment messages. We have used synchronous message channels for showing the communication between two processes which are presented as devices in our model. The processes in our model used dummy data objects which are shown as 0 in the simulation results in Figure 21. The simulation result is not complete in the figure because the simulation runs continuously as the devices always stay alive in real-life scenarios.

The huge deployment of future IoT devices in every sector of our society needs distributed and decentralized identity management and secure communication to ensure the confidentiality, integrity, privacy as well as service availability. The above informal analysis of secure communication, identity management model performance, and formal verification results using Scyther and SPIN tool show that our approach for distributed and decentralized identity management and secure communication model can be implemented for the deployment of secure communication and identity management of resource-constrained IoT devices. In the next section, we have the final discussion and concluding comments.

## 6. Discussion

The huge deployment of IoT infrastructure is an ongoing work. Current researches are focused on innovative ideas for new IoT applications. On the other hand, there is less emphasis on studies for secure IoT data communication and processing. Data collected from authenticated sources should only be processed because, if data from unauthenticated sources are used for decision-making, it could be harmful and misleading. Edge/fog computing for data processing is getting popular as it can offer data processing near the data sources and provide results with quick response and low latency. As the data is processed at fog/edge devices in a distributed manner, and the data source’s identity is worthy, it is also essential to provide identity solutions at the fog/edge layer and not consider centralized identity, authentication authorization methods. For smooth communication between devices trust is essential. Figure 3 shows that besides other parameters, identity management, device authentication, authorization, and security key management play vital roles in the enhancement of IoT trust management. Identity-based trust management is one of several trust management. Only identity-based trust management is not enough for the IoT infrastructure as it is built upon heterogeneous devices connected via the internet. We will address different others’ trust management challenges and mechanisms in our future work. Additionally, the results of IoT based solutions are for the wellbeing of human society and innovative IoT applications connect the user applications with end devices, it is involved in the processing of private data from the society. The privacy of the data generated by IoT devices also needs to be considered while addressing identity management.

## 7. Conclusions

In this paper, we have presented an extensive survey on IoT identity management solutions with detailed discussion on their strengths and weaknesses. We have thoroughly described IoT authentication and authorization and identity management processes (Figure 6). A novel distributed decentralized IoT identity management architecture (Figure 7, Figure 8 and Figure 9) with authentication (Figure 10 and Figure 11), authorization (Figure 10), secure communication (Figure 8 and Figure 9), and key distribution protocol (Figure 13) is proposed. The verification results (Figure 16, Figure 17 and Figure 18) show our proposed secure communication protocol’s performance. Figure 19, Figure 20 and Figure 21 present the validation of our proposed model. Our proposed model is location-based identity management (Figure 7). The location information is one of the crucial parameters while considering the privacy of data generated from human societies. We will address privacy issues related to IoT data and identity management in our future work. We will extend this work in our future research with experiments to observe our model’s influence on power management and include statistical analysis, representing our model’s performance evaluation.

## Figures and Tables

**Table 1:** Different terms notation and descriptions table.

Notation	Description
IoTD	IoT Device
EIoTG	Edge IoT Gateway
LIdP	Local Identity Provider
GIdP	Global Identity Provider
ID_IoTD_, ID_EIoTG_, ID_LIdP_, ID_GIdP_	Identities of IoTD, EIoTG, LIdP and GIdP
Pbk_EIoTG_, PbK_LIdP_, PbK_GIdP_	Public keys of EIoTG, LIdP and GIdP
Prk_EIoTG_, PrK_LIdP_, PrK_GIdP_	Private keys of EIoTG, LIdP and GIdP
SrK_IoTD-EIoTG_	Shared key for IoTD and EIoTG
Sk_IoTD-EIoTG_	Session key for IoTD and EIoTG

**Table 2:** Comparative analysis of different features of identity management solutions.

Features ^1^	Paper References (Discussed)	Paper References (Not Discussed)	IMSC-EIoTD (Our Approach)
Security	[31,32,41,42,44,45,46,47,48,49]	[43]	Addressed
Privacy	[31,32,42,44,45]	[41,43,46,47,48,49]	Discussed
Trustworthiness	[31,46,49]	[32,41,42,43,44,45,46,47,48]	Described
Mobility	[31,43,44,45,47]	[32,41,42,46,48,49]	Addressed
Usability	[45,46]	[31,32,41,42,43,44,47,48,49]	Discussed
Affordability		[31,32,41,42,43,44,45,46,47,48,49]	Discussed
Law enforcement		[31,32,41,42,43,44,45,46,47,48,49]	Discussed
Interoperability	[31,44,45,46,47,48,49]	[32,41,42,43]	Discussed
Functionality	[31,44,45,46,47,49]	[32,41,42,43,48]	Described
Scalability	[31,41,44,48]	[32,42,43,45,46,47,49]	Addressed
Administration	[31,45,49]	[32,41,42,43,44,46,47,48]	Described

^1^ Features are considered from the performance evaluation criteria discussed above.
